# Unraveling Mitochondrial Genome Evolution in *Puccinia striiformis* f. sp. *elymi*, the Elymus Stripe Rust Fungus

**DOI:** 10.3390/jof12030217

**Published:** 2026-03-18

**Authors:** Yi Wu, Hai Xu, Shuwaner Wang, Yue Xiao, Xin Li, Suizhuang Yang, Xinli Zhou, Chongjing Xia

**Affiliations:** Wheat Research Institute, Engineering Research Center of Biomass Materials, Ministry of Education, College of Life Sciences and Agri-Forestry, Southwest University of Science and Technology, Mianyang 621010, China; 17828285566@swust.edu.cn (Y.W.); xuhai@mails.swust.edu.cn (H.X.); wangshuwaner@mails.swust.edu.cn (S.W.); xiaoyuejy101@mails.swust.edu.cn (Y.X.); wheat4u@swust.edu.cn (X.L.); yangszh@126.com (S.Y.)

**Keywords:** *Puccinia striiformis* f. sp. *elymi*, mitochondrial genome, PacBio HiFi sequencing, intron loss, host specialization, rust fungi, phylogenomics

## Abstract

*Puccinia striiformis* f. sp. *elymi* (*Pse*) is a specialized forma specialis of stripe rust infecting *Elymus dahuricus*, yet its mitochondrial evolution remains poorly understood. In this study, we assembled the complete mitogenome of *Pse* using PacBio HiFi sequencing, yielding a circular mitogenome of 72,952 bp. This reveals a striking asymmetric evolutionary pattern with a 28.34% genomic contraction compared to the wheat stripe rust *P. striiformis* f. sp. *tritici* (*Pst*-CYR32). Our analysis demonstrates that this streamlining is strictly driven by a massive and systematic loss of mitochondrial introns. The *Pse* mitogenome exhibits negative GC-skew (−0.0184) consistent with strand-asymmetric mutational pressure, while maintaining a strictly conserved and syntenic complement of all 14 core protein-coding genes (PCGs), alongside 24 tRNAs and 2 rRNAs. Phylogenomic analysis positions *Pse* as sister to the *Pst* clade with strong support (100% bootstrap). A 748-bp SNP cluster within *nad4* (14.2% sequence divergence versus 3.1% genome-wide average) provides a candidate molecular marker for lineage differentiation, pending population-level validation. This study establishes a genomic foundation for investigating mitochondrial reductive evolution in host-specialized rust lineages, highlighting the dynamic role of introns in driving organellar genome size variation.

## 1. Background

*Puccinia striiformis* Westend. (stripe rust fungus) represents a species complex comprising multiple host-specialized formae speciales that infect diverse Poaceae hosts globally [1,2]. While *P. striiformis* f. sp. *tritici* (*Pst*) is a devastating pathogen of wheat (*Triticum aestivum*), *P. striiformis* f. sp. *elymi* (*Pse*) exhibits strict specialization toward *Elymus* species (wild rye grasses), including the economically important forage grass *Elymus dahuricus* [3,4]. Despite morphological similarities, molecular evidence indicates that *Pse* and *Pst* represent distinct evolutionary lineages within the *P. striiformis* complex, with host range constituting a primary criterion for their taxonomic differentiation [5,6].

The *P*. *striiformis* species complex serves as an exceptional framework for investigating host-driven speciation among obligate biotrophic fungi. While *Pst* has evolved to exploit the genetically uniform landscapes of modern cultivated wheat, *Pse* maintains a highly specialized parasitic relationship with heterogeneous populations of wild rye grasses, such as *Elymus* dahuricus. This rigid host specialization implies that the divergence between *Pse* and *Pst* is not merely a superficial shift in pathogenicity but rather a profound evolutionary trajectory driven by distinct ecological niches. The transition between wild ecological systems and intensive agricultural environments imposes contrasting selective pressures on the pathogen. Consequently, exploring the genomic signatures of these divergent lineages is essential for understanding how host adaptation influences the architectural evolution of both nuclear and organellar genomes.

Mitochondrial genomes (mitogenomes) in fungi typically encode a conserved core set of 14 respiratory protein-coding genes (PCGs), namely, *atp6*, *atp8*, *atp9*, *cob*, *cox1-3*, and *nad1-6* plus *nad4L*, alongside rRNA and tRNA genes [7]. However, rust fungi (*Pucciniales*) display remarkable variation in mitogenome architecture. Extensive comparative studies reveal sizes ranging from approximately 32 kb in *Phakopsora* species [8] to 93 kb in *Austropuccinia psidii* [9], with intermediate sizes (~77–102 kb) observed across *Puccinia* species [10,11]. This variation primarily reflects differences in intron content, homing endonuclease gene (HEG) proliferation, and intergenic spacer expansion rather than in core gene repertoire [9,12]. HEGs act as selfish genetic elements that promote the mobility of their host introns, and their accumulation frequently drives mitochondrial genome expansion in fungi. Within this evolutionary context, the extreme size variation documented in rust fungal mitogenomes highlights a remarkably dynamic pattern of organellar evolution. This profound architectural plasticity is not driven by changes in the core respiratory machinery but rather by the differential accumulation, proliferation, or eradication of mobile genetic elements. Specifically, group I and group II introns, along with their associated HEGs, act as classic “selfish genetic elements”. HEGs encode highly specific nucleases that facilitate their own lateral mobility and introgression into intronless alleles, thereby ensuring their persistence and spread within a population independently of the host’s organismal fitness. In expanding mitogenomes, such as that of *A. psidii*, the unchecked proliferation of these selfish elements, combined with the subsequent expansion of intergenic spacers, leads to significant genomic bloating. Conversely, fungal lineages subjected to rigorous metabolic constraints or distinct population bottlenecks may experience selective pressures favoring genomic economy, resulting in the systematic purging of these mobile elements [13]. Notably, all previously characterized rust mitogenomes retain the complete set of 14 core respiratory genes, underscoring their functional indispensability for oxidative phosphorylation [7,8].

The relationship between mitogenome architecture and host adaptation in obligate biotrophic pathogens remains poorly understood. While nuclear genome evolution clearly drives host specialization in rust fungi [5], the contribution of organellar genome reorganization requires careful evaluation to distinguish adaptive signatures from neutral evolutionary processes [14]. Comparative mitogenomic analyses therefore provide valuable insights into lineage-specific evolutionary trajectories while avoiding overinterpretation of correlative patterns.

Here, we report a complete mitogenome sequence of *P. striiformis* f. sp. *elymi* generated using PacBio HiFi sequencing technology. Through comparative analysis with wheat-infecting *P*. *striiformis* isolates and related rust species, we characterize the structural features underlying its reduced genome size, explore the evolutionary dynamics of its introns within this extremely streamlined mitogenome, and assess the phylogenetic position of *Pse* within the *P*. *striiformis* complex. This study establishes a genomic foundation for investigating mitochondrial reductive evolution in host-specialized rust lineages, highlighting the dynamic role of introns in driving organellar genome size variation.

## 2. Materials and Methods

### 2.1. Sample Collection and Phenotypic Validation

**Figure 1:**
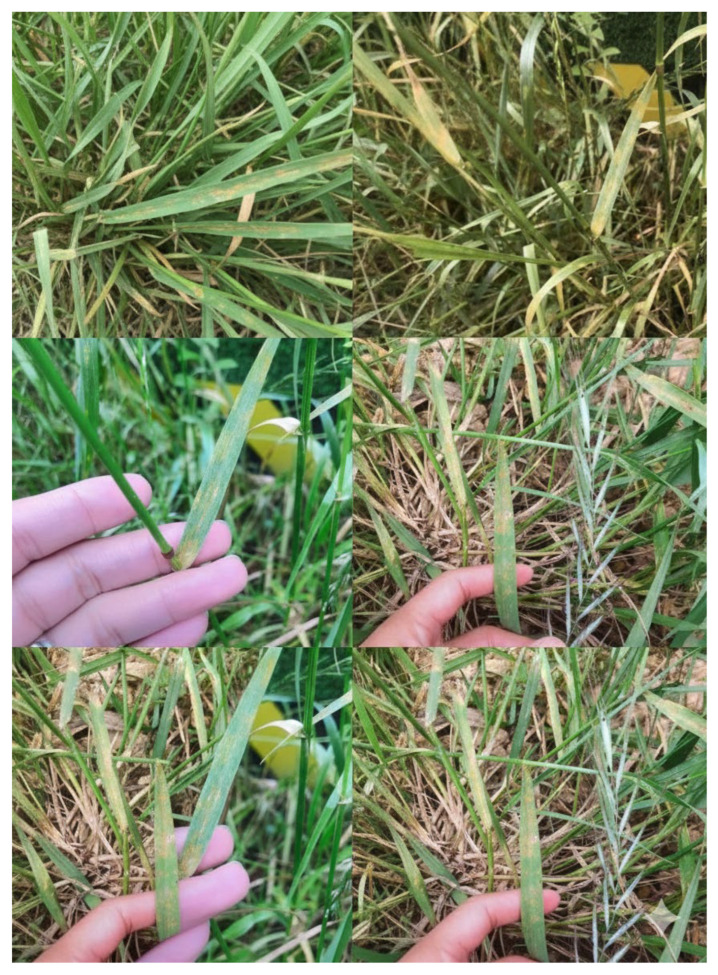
Pathological symptoms and host specialization of *Puccinia striiformis* f. sp. *elymi* (*Pse*). Typical symptoms observed on *Elymus dahuricus* leaves at 14 days post-inoculation with *Pse*. The image displays characteristic orange-yellow uredinia arranged in necrotic stripes along the leaf veins, indicative of high virulence (Infection Type 4). Parallel inoculation on the susceptible wheat cultivar ‘Mingxian 169’ resulted in a complete lack of symptoms (not shown), confirming the specialized pathological identity and strict host range of the isolate used for mitogenomic sequencing.

An extensive bulk collection of infected Elymus dahuricus was conducted across four geographical locations in Mianyang, Sichuan Province, China (31.53° N, 104.68° E). From this collection, a representative *Puccinia striiformis* f. sp. *elymi* (*Pse*) isolate (Figure 1) was purified via three rounds of single-pustule isolation. Freshly collected urediniospores from this purified isolate were utilized for all subsequent inoculations. Host specificity was rigorously validated through multiple independent biological replicates of reciprocal inoculation assays on seedlings (at the one-leaf-one-heart stage) of *E. dahuricus* and Triticum aestivum (cv. ‘Mingxian 169’). At 14 days post-inoculation, *Pse* produced typical stripe rust symptoms on *E. dahuricus*, characterized by orange-yellow uredinia arranged in necrotic stripes, representing a high virulence level (IT 4). In contrast, the *Pse* isolate was completely avirulent (IT 0) on the susceptible wheat cultivar ‘Mingxian 169’, showing no observable symptoms.

### 2.2. DNA Extraction and PacBio HiFi Sequencing

Total genomic DNA was extracted from a single infected leaf with urediniospores using the CTAB method. Briefly, 10 mg of frozen spores were ground to a fine powder under liquid nitrogen and incubated in 700 µL CTAB buffer (2% CTAB, 100 mM Tris-HCl, pH 8.0, 20 mM EDTA, 1.4 M NaCl, 1% β-mercaptoethanol) at 65 °C for 45 min with gentle inversion every 10 min; this was followed by chloroform:isoamyl alcohol (24:1) extraction. DNA was precipitated with isopropanol, washed twice with 70% ethanol, and resuspended in TE buffer. DNA integrity was verified by pulsed-field gel electrophoresis, and purity was assessed using a NanoDrop One spectrophotometer (Thermo Fisher Scientific, Waltham, MA, USA; A260/A280 = 1.87; A260/A230 = 2.15). DNA concentration was quantified with a Qubit 4 Fluorometer (Thermo Fisher Scientific). High-molecular-weight DNA was sheared into 15–20 kb fragments and sequenced on the PacBio Revio platform (Pacific Biosciences, Menlo Park, CA, USA) in Novogene (Beijing, China). Circular Consensus Sequencing (CCS) was employed to generate high-fidelity (HiFi) reads with a median read accuracy of ≥Q40 (99.99%), ensuring precise base-calling for mitochondrial variant identification [15].

### 2.3. Mitogenome Assembly and Structural Verification

The *Pse* mitogenome was assembled de novo using GetOrganelle v1.7.7.0 [16], yielding a single circular contig of 72,952 bp. The circular topology was independently verified using Canu v2.2 [17]. Assembly integrity was validated by mapping raw PacBio HiFi reads back to the consensus sequence using pbmm2 v1.10.0 [15], demonstrating uniform sequencing depth and continuous read coverage across all gene junctions. The intergenic spacer between *ND3* and *ATP8* was precisely measured to ensure no assembly gaps or misalignments were present in this contracted region.

#### Nucleotide Composition and Homology Search

Nucleotide composition and strand asymmetry were analyzed using BioEdit v7.2 and custom Python3.13.5 scripts. GC and AT skews were calculated using the following formulas: GC-skew = (G − C)/(G + C) and AT-skew = (A − T)/(A + T).

To assess the protein-coding gene (PCG) repertoire, a multi-step homology search was conducted using the 14 core PCGs from *Pst*-CYR32 (MN746374) as reference protein sequences. Initial BLASTn and BLASTx (version 2.17.0+) searches against the *Pse* assembly successfully identified all 14 core orthologous PCGs.

### 2.4. Functional Annotation and Phylogenetic Analysis

Functional annotation was performed using MITOS2 [18] (Translation Table 4), with gene boundaries manually refined through BLASTp (version 2.17.0+) searches. tRNA genes were identified via tRNAscan-SE v2.0 [19], and rRNA genes were localized through alignment with reference rust mitogenomes. To ensure the highest accuracy of the functional annotation, a rigorous dual-verification mechanism was implemented following the initial automated predictions. While automated pipelines like MITOS2 provide a robust structural framework, they can occasionally misidentify precise start and stop codons in fungal mitogenomes due to the idiosyncratic use of alternative genetic codes and abbreviated termination codons. Therefore, the boundary of each of the 14 core protein-coding genes (PCGs) was subjected to exhaustive manual curation. This involved extracting the translated amino acid sequences and aligning them against curated reference orthologs from the *P. striiformis* complex, allowing for the precise visual confirmation of translation initiation and termination sites. Furthermore, the mapping of tRNA genes served as a critical structural checkpoint. The secondary structure predictions generated by tRNAscan-SE v2.0 were utilized not only to confirm tRNA identity but also to define absolute non-coding boundaries, ensuring that no spurious overlaps existed between the predicted tRNA cloverleaf structures and adjacent PCG open reading frames. The final circular map was visualized using OGDraw v1.3.1 [20].

For phylogenetic reconstruction, shared PCG amino acid sequences were concatenated, aligned with MAFFT (v7.505; with default parameters) [21], trimmed by trimAl (version 1.4.rev15) [22], and used for supermatrix construction via AMAS. A partitioned ML tree was generated in IQ-TREE2 (version 2.2.0) [23], with an optimal evolutionary model selected by ModelFinder Plus and topology reliability validated by 1000 bootstraps.

Following the finalization of the structural and functional annotations, the data was strictly formatted to meet the rigorous curation standards required by the NCBI GenBank database. This necessitated the generation of highly specific submission files, primarily encompassing a pristine nucleotide sequence file in FASTA format (.fsa) and a corresponding five-column feature table (.tbl). The .tbl file was meticulously constructed and manually audited line by line to encapsulate all biological features, verifying the exact coordinates of exons, the few remaining introns, and intergenic spacers. This manual auditing ensured that all conceptual translation products generated by Translation Table 4 exactly matched the verified PCGs without internal stop codons or frameshifts. Subsequent processing utilized NCBI’s command-line utilities to compile these components into a finalized submission archive. Only after passing these stringent internal validation checks was the complete, error-free mitogenome sequence deposited into the GenBank repository, culminating in the assignment of the accession number PZ137757.

### 2.5. Phylogenetic Reconstruction

A comparative dataset was constructed using five reference mitogenomes: *Pst* (MN746374, NC039655), *Pt* (MN004749), *Pgt* (NW003526743), and *Ap* (NC044121, as the outgroup). Phylogenetic reconstruction was based on the 14 orthologous PCGs shared by all species. Protein sequences were aligned using MAFFT v7.505 and concatenated via Phylosuite v1.2.3. The Maximum Likelihood (ML) tree was generated using IQ-TREE v2.2.0 with 1000 ultrafast bootstrap replicates. The tree was rooted with *A. psidii* and visualized in iTOL v6.

### 2.6. Variant Analysis and SNP Cluster Identification

The *Pse* mitogenome was aligned against *Pst*-*CYR32* using nucmer (MUMmer v4.0). Single Nucleotide Polymorphisms (SNPs) were extracted and verified by re-mapping raw HiFi reads to ensure 100% allelic consistency. Analysis focused on a localized cluster of substitutions within the *nad4* gene, which was evaluated as a candidate molecular signature for distinguishing *Pse* from *Pst* lineages.

## 3. Results

### 3.1. Host Specificity of Pse

As detailed in Section 2, reciprocal inoculation assays validated the strict host specificity of the *P. striiformis* f. sp. *elymi* isolate. The clear pathological differentiation (high virulence on *E. dahuricus* and complete avirulence on wheat) provided the biological foundation for comparative mitogenomic analysis against other rust fungi.

### 3.2. Mitogenome Architecture and Nucleotide Composition

**Figure 2:**
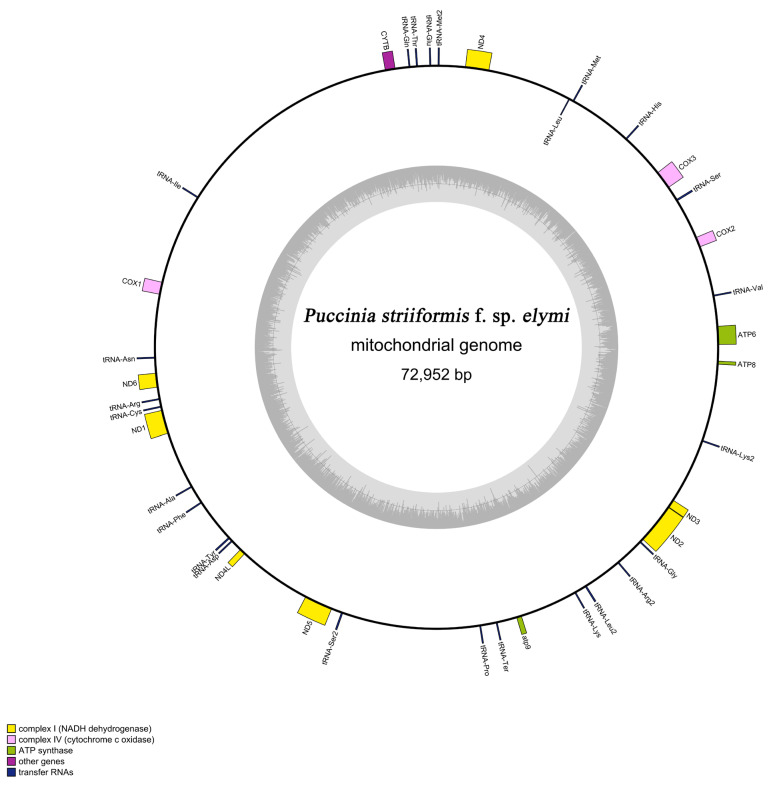
The map illustrates the 72,952 bp circular molecule, highlighting the complete and highly conserved set of 14 core protein-coding genes.

The *Pse* mitogenome was assembled as a single circular molecule of 72,952 bp with a mean GC content of 34.2% (Figure 2; Table 1). Annotation identified 40 functional elements: 14 core protein-coding genes (PCGs), 24 tRNA genes, and 2 rRNA genes (*rnl*, *rns*). All annotated genes reside on the same DNA strand and are transcribed unidirectionally (clockwise), a conserved feature across *Pucciniales* [8,9].

Strand-specific nucleotide bias analysis revealed a negative GC-skew (−0.0184) and near-neutral AT-skew (−0.0001). This asymmetry aligns with the strand-displacement model of mitochondrial replication, wherein prolonged single-stranded exposure of the lagging strand promotes C→T deamination [12,24]. Gene density (5.21 genes per 10 kb) exceeds that of *Pst*-CYR32 (3.79 genes per 10 kb) but remains within the natural variation observed across rust fungi (Table 1) [8,9].

### 3.3. Genome Size Variation and Intron Dynamics

A meticulous examination of the comparative metrics presented in Table 1 reveals a pronounced divergence in mitogenome architecture among the analyzed rust lineages. The absolute size of the *Pse* mitogenome is resolved at 72,952 bp. When directly juxtaposed with the wheat-infecting *P*. *striiformis* f. sp. *tritici* strains, a substantial and precise genomic contraction is evident; specifically, the *Pse* mitogenome is 28,861 bp smaller than that of *Pst*-*DK0911* (101,813 bp) and 28,569 bp smaller than that of *Pst*-*CYR32* (101,521 bp). To further contextualize this structural streamlining, *Pse* also exhibits a more compact architecture than other primary wheat pathogens, including *P*. *triticina* isolate HnZU18-3 (77,894 bp) and *P*. *graminis* f. sp. *tritici* isolate *CRL75*-36-700-3 (79,748 bp). Furthermore, it is markedly smaller than the outgroup *Austropuccinia psidii* isolate MF-1, which possesses a genome size of 93,299 bp.

Crucially, an in-depth dissection of the gene repertoires across these lineages exposes a compelling inverse relationship between total genome size and the retention of core respiratory elements. Intuitively, massive genomic expansions might be expected to correlate with enhanced or preserved genetic complexity. However, the empirical data contradict this assumption. Despite their expansive sizes exceeding 101 kb, both *Pst*-*DK0911* and *Pst*-*CYR32* have experienced gene loss, retaining only 13 core protein-coding genes (PCGs). This phenomenon is similarly observed in the large 93.2 kb genome of *A. psidii*, which encodes a mere 12 core PCGs. In stark contrast, the radically streamlined 72.9 kb mitogenome of *Pse* successfully harbors the complete complement of all 14 core PCGs. This evolutionary strategy of maximal functional retention within a minimal physical footprint is not entirely unique to *Pse*; it is mirrored by *P*. *triticina* (*Pt*-HnZU18-3), which also maintains all 14 core PCGs within its relatively compact 77.9 kb genome, whereas *P*. *graminis* (*Pgt*-CRL75, 79.7 kb) possesses 13 PCGs. This detailed juxtaposition demonstrates unequivocally that the massive size discrepancies do not confer an advantage in core respiratory coding capacity; instead, the *Pse* lineage has achieved a highly economical architecture, aggressively purging non-essential sequences while fiercely guarding the functional integrity of its oxidative phosphorylation machinery.

The *Pse* mitogenome (72.9 kb) represents a 28.3% reduction relative to *Pst*-CYR32 (101.8 kb). However, this contraction must be contextualized within the broader size spectrum of rust fungal mitogenomes, which ranges from 31.8 kb (*P. pachyrhizi*) to 93.3 kb (*A. psidii*) [8,9]. Mitogenome size of *Pse* falls within the intermediate range observed for *Puccinia* species (77.9–101.8 kb), indicating moderate—not extreme—streamlining.

**Figure 3:**
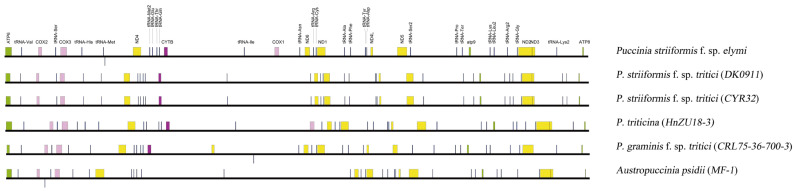
Colored blocks represent conserved gene clusters, while white gaps indicate the massive and systematic loss of mitochondrial introns in *Pse*. Note the strict syntenic conservation of all 14 core respiratory genes across the analyzed lineages.

Size reduction primarily reflects systematic intron loss rather than core gene deletion. The *cox1* gene in *Pse* spans 1581 bp compared to 10,234 bp in *Pst*-CYR32, representing an 84.5% reduction attributable to the loss of 11 introns (Figure 3). Similarly, *cob* decreased from 6825 bp (*Pst*) to 1164 bp (*Pse*) through the loss of 4 introns. Across the mitogenome, *Pse* retains only 2 introns (both in *cox1*) versus 11–12 introns in *Pst* isolates [10,25]. This pattern of reductive evolution parallels observations in other fungal lineages where intron loss correlates with reduced homing endonuclease gene (HEG) content [11,12].

Intergenic spacer length also contracted from 18.7 kb in *Pst*-CYR32 to 12.3 kb in *Pse*. Notably, non-coding ORFs (ncORFs) were absent from the *Pse* mitogenome, contrasting with *A. psidii,* where 33 ncORFs (45% of total genes) drive genome expansion [9]. These findings indicate that mitogenome size variation in rust fungi stems primarily from differential accumulation of mobile elements and introns rather than core gene repertoire changes [8,9,11].

### 3.4. Phylogenomic Placement of Pse Within Pucciniales Orthologous PCGs

**Figure 4:**
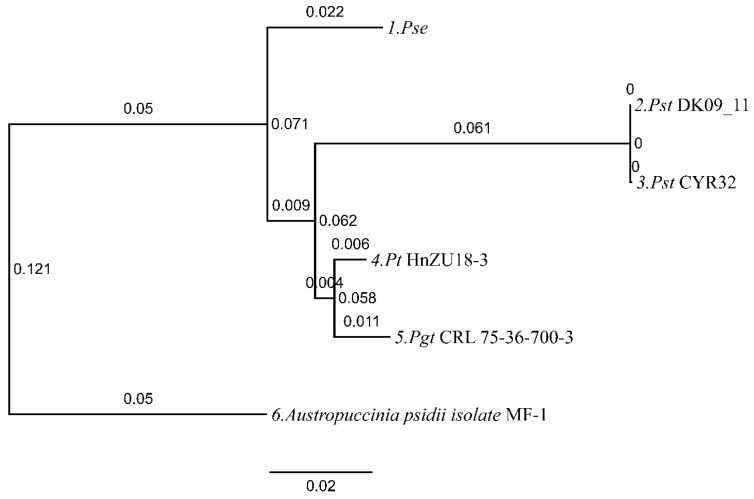
Maximum-likelihood phylogenetic tree of *Pse* and related rust species based on 12 orthologous mitochondrial protein-coding genes.

Maximum-likelihood phylogeny based on concatenated alignments of 14 shared mitochondrial PCGs positioned *Pse* as sister to the *Pst* clade with 100% bootstrap support (Figure 4). This topology corroborates previous nuclear genome-based phylogenies (e.g., using ITS and other conserved nuclear markers), confirming *Pse* and *Pst* as distinct but closely related lineages within the *P. striiformis* complex [4,5,6]. The strict conservation of tRNA gene complement (24 genes) and rRNA architecture across all *Puccinia* species contrasts with the dynamic evolution of intron content and mobile elements, underscoring differential evolutionary rates among mitogenome components [8,9].

### 3.5. Identification of a Lineage-Specific SNP Cluster in nad4

**Figure 5:**
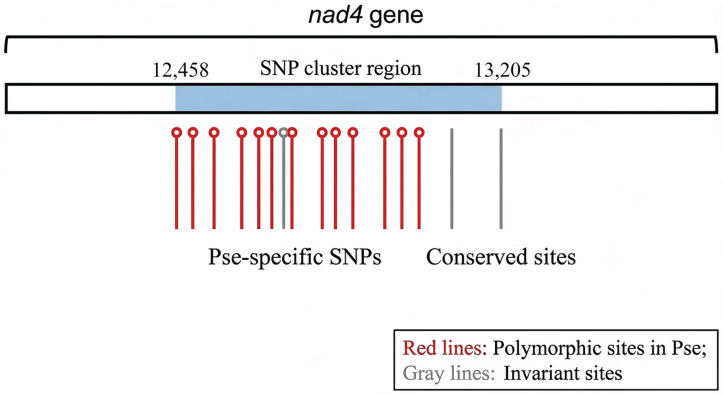
Visual alignment of the conserved SNP cluster within the nad4 gene for *Pse* identification. The 748-bp segment (coordinates 12,458–13,205) highlights the high-density substitutions unique to *Pse*. Note: This SNP cluster was cross-verified against both *Pst*-*CYR32* and *Pst*-*93*-*210* reference genomes to ensure its stability as a lineage-specific signature.

Comparative alignment identified a 748-bp region within *nad4* (coordinates 12,458–13,205) exhibiting elevated nucleotide divergence between *Pse* and *Pst* lineages (14.2% sequence divergence versus 3.1% genome-wide average). This cluster contains 17 fixed SNPs, 11 of which result in non-synonymous substitutions within predicted transmembrane domains of Complex I (Figure 5). While preliminary, this signature provides a candidate molecular marker for distinguishing *Pse* from *Pst* in diagnostic assays. Validation across geographically diverse *Pse* isolates remains necessary before diagnostic application [26].

## 4. Discussion

### 4.1. Mitogenome Size Variation Within the Natural Spectrum of Rust Fungi

The *Pse* mitogenome (72.9 kb) represents a 28.3% reduction relative to that of *Pst*-CYR32 (101.8 kb). However, this contraction must be interpreted within the broader context of mitogenome size variation across *Pucciniales*. Rust fungal mitogenomes span a remarkable range from 31.8 kb (*Phakopsora pachyrhizi*) to 93.3 kb (*Austropuccinia psidii*) [8,9], with intermediate sizes observed in *P. graminis* (79.7 kb) and *P. triticina* (77.9 kb) [10]. Mitogenome size of *Pse* falls within this natural continuum rather than representing an “extreme” outlier. This spectrum reflects differential accumulation of mobile genetic elements rather than fundamental alterations to core respiratory machinery [9,12].

Size reduction in *Pse* is strictly driven by massive and systematic intron loss, while the complete repertoire of 14 core respiratory genes remains highly conserved and syntenic. For instance, the *cox1* gene contracted from 10,234 bp in *Pst* to 1581 bp in *Pse* through the loss of 11 introns, a pattern consistent with reductive evolution documented across diverse fungal lineages [11,27]. Similarly, *cob* decreased from 6825 bp to 1164 bp via the loss of four introns. These observations align with the established principle that intron dynamics—not core gene repertoire—drive mitogenome size variation in fungi [8,9,12]. Notably, *A. psidii* exemplifies the opposite trajectory: its 93.3-kb genome harbors 33 nonconserved ORFs (45% of total genes), many within introns, demonstrating how HEG proliferation can expand mitogenome size [9,27].

### 4.2. Intron Loss as a Driver of Mitogenome Streamlining

The *Pse* mitogenome exhibits significant streamlining, characterized by a 28.34% contraction strictly driven by a systematic loss of introns. This genomic architecture, achieved without the loss of any core respiratory genes, provides compelling evidence that intron dynamics—rather than core gene repertoire—are the primary drivers of mitogenome size variation in rust fungi. This parallels patterns observed in other streamlined fungal mitogenomes [11,12]. This reductive evolution likely reflects secondary loss of homing endonuclease genes (HEGs), which normally maintain intron persistence through mobility mechanisms [11,28]. Absence of LAGLIDADG or GIY-YIG motifs within *Pse* introns—contrasting with nine such elements in *A. psidii* [9]—supports this interpretation. Without HEG-mediated mobility, introns become vulnerable to deletion via slipped-strand mispairing or microhomology-mediated repair [11].

The negative GC-skew (−0.0184) observed in *Pse* may reflect strand-asymmetric mutational pressure during replication [24], potentially accelerating purging of non-essential sequences. However, GC-skew alone cannot prove replication mechanism causality; many fungi exhibit strand asymmetry without invoking adaptive explanations [14]. We therefore interpret reduced intron content as a neutral or mildly adaptive outcome of HEG loss rather than strong selection for replication efficiency.

### 4.3. Diagnostic Potential of the nad4 SNP Cluster

The identification of a 748-bp SNP cluster within the nad4 gene, exhibiting a 14.2% sequence divergence compared to the 3.1% genome-wide average, provides a compelling candidate molecular marker for distinguishing the *Pse* and *Pst* lineages. The *nad4* gene encodes a core subunit of the mitochondrial respiratory Complex I (NADH:ubiquinone oxidoreductase). Remarkably, 11 of these lineage-specific non-synonymous substitutions map directly to the predicted transmembrane domains of this complex. Since the transmembrane helices of Nad4 are intricately involved in the proton translocation mechanism, such a dense cluster of amino acid alterations could theoretically influence the structural conformation of the proton-pumping module. While these mutations clearly do not compromise essential respiratory viability—given the pathogen’s high fitness on its specific host—they warrant future biochemical investigations to determine if they represent adaptive modifications to distinct ecological niches or merely reflect neutral genetic drift following speciation.

Translating this genomic signature into a robust diagnostic tool necessitates stringent, large-scale population-level validation. Relying solely on the reference mitogenomes of single isolates carries the inherent risk of overgeneralizing a local, transient polymorphism as a fixed lineage-specific marker, particularly given the well-documented intraspecific mitogenome variation in rust fungi. Therefore, comprehensive screening across geographically diverse *Pse* and *Pst* populations is mandatory before field application [29].

If empirically validated across diverse populations, mitochondrial markers like this *nad4* cluster offer profound analytical advantages. Unlike single-copy nuclear genes, the mitochondrial genome exists in exceptionally high copy numbers within a single fungal cell. In complex agricultural environments, where wild *Elymus* grasses often co-exist near cultivated wheat fields, epidemiological monitoring frequently requires detecting trace amounts of pathogen DNA in asymptomatic tissues or environmental spore traps. An mtDNA-based molecular assay (such as LAMP or multiplex qPCR) would leverage this inherent biological amplification, providing the superior sensitivity required to reliably detect and discriminate mixed infections even at critical, low-biomass thresholds.

### 4.4. Conclusions

This study reports the first complete mitogenome of *Puccinia striiformis* f. sp. *elymi* (72.9 kb), assembled via PacBio HiFi sequencing. Comparative analysis reveals that the reduced mitogenome size of *Pse* falls within the natural variation observed across Pucciniales (31.8–93.3 kb) [8,9]. We demonstrate that this structural streamlining is strictly driven by massive and systematic intron loss, while maintaining a perfectly conserved and syntenic set of all 14 core respiratory genes. Phylogenomic analysis positions *Pse* as sister to the *Pst* clade with strong support. A SNP cluster within *nad4* provides a preliminary candidate marker for lineage differentiation, though population-level validation is required before diagnostic application. Our findings highlight the dynamic role of mobile elements, rather than core gene evolution, in shaping organellar genome architecture during rust fungi speciation.

## Figures and Tables

**Table 1:** Comparison of mitochondrial genome features among *P. striiformis* f. sp. *elymi* and other related rust fungi.

Species/Isolate	Size (bp)	PCGs *	tRNAs	NCBI Accession No.
*P. striiformis* f. sp. *elymi* (*Pse*)	72,952	14	25	Current Study
*P. striiformis* f. sp. *tritici* (*Pst*-DK0911)	101,813	13	25	MN746374
*P. striiformis* f. sp. *tritici* (*Pst*-CYR32)	101,521	13	25	NC039655
*P. triticina* (*Pt*-HnZU18-3)	77,894	14	25	MN004749
*P. graminis* f. sp. *tritici* (*Pgt*-CRL75)	79,748	13	24	NW003526743
*Austropuccinia psidii* (MF-1)	93,299	12	24	NC044121

* PCGs = protein-coding genes encoding core respiratory subunits (14 conserved: *atp6*, *atp8*, *atp9*, *cob*, *cox1–3*, *nad1–6*, *nad4L*).

## Data Availability

The assembled and annotated mitogenome is deposited in NCBI GenBank under accession number PZ137757.
